# A General Mathematical Algorithm for Predicting the Course of Unfused Tetanic Contractions of Motor Units in Rat Muscle

**DOI:** 10.1371/journal.pone.0162385

**Published:** 2016-09-13

**Authors:** Rositsa Raikova, Piotr Krutki, Jan Celichowski

**Affiliations:** 1 Institute of Biophysics and Biomedical Engineering, Bulgarian Academy of Sciences, Sofia, Bulgaria; 2 Department of Neurobiology, University School of Physical Education, Poznań, Poland; Duke University, UNITED STATES

## Abstract

An unfused tetanus of a motor unit (MU) evoked by a train of pulses at variable interpulse intervals is the sum of non-equal twitch-like responses to these stimuli. A tool for a precise prediction of these successive contractions for MUs of different physiological types with different contractile properties is crucial for modeling the whole muscle behavior during various types of activity. The aim of this paper is to develop such a general mathematical algorithm for the MUs of the medial gastrocnemius muscle of rats. For this purpose, tetanic curves recorded for 30 MUs (10 slow, 10 fast fatigue-resistant and 10 fast fatigable) were mathematically decomposed into twitch-like contractions. Each contraction was modeled by the previously proposed 6-parameter analytical function, and the analysis of these six parameters allowed us to develop a prediction algorithm based on the following input data: parameters of the initial twitch, the maximum force of a MU and the series of pulses. Linear relationship was found between the normalized amplitudes of the successive contractions and the remainder between the actual force levels at which the contraction started and the maximum tetanic force. The normalization was made according to the amplitude of the first decomposed twitch. However, the respective approximation lines had different specific angles with respect to the ordinate. These angles had different and non-overlapping ranges for slow and fast MUs. A sensitivity analysis concerning this slope was performed and the dependence between the angles and the maximal fused tetanic force normalized to the amplitude of the first contraction was approximated by a power function. The normalized MU contraction and half-relaxation times were approximated by linear functions depending on the normalized actual force levels at which each contraction starts. The normalization was made according to the contraction time of the first contraction. The actual force levels were calculated initially from the recorded tetanic curves and subsequently from the modeled curves obtained from the summation of all models of the preceding contractions (the so called “full prediction”). The preciseness of the prediction was verified by two coefficients estimating the error between the modeled and the experimentally recorded curves. The proposed approach was tested for 30 MUs from the database and for three additional MUs, not included in the initial set. It was concluded that this general algorithm can be successfully used for modeling of a unfused tetanus course of a single MU of fast and slow type.

## Introduction

The mechanical output of a skeletal muscle is the sum of forces generated by its active motor units (MUs), and the forces of the individual MUs depend on a firing pattern of the motoneurons. During voluntary activity, motoneurons generate trains of action potentials at variable time intervals [1–5], and each of these action potentials evokes a twitch-like force response [6–8]. However, when MU tetanic contractions evoked by trains of stimuli at variable time intervals (which cause considerable force fluctuations) are decomposed into twitch-shape responses to successive stimuli, the amplitude and the time parameters of these responses appear to be highly variable, and considerable differences in the tetanic force development are observed between the three types of MUs [9, 10]. For slow (S) MUs, individual twitch-like responses have force amplitudes up to seven times higher and contraction and relaxation phases over three times longer in comparison to the single twitch [11]. For fast resistant-to-fatigue (FR) MUs, variability of the twitch parameters is in general much lower (force amplitudes up to three times higher than the single twitch), whereas for some fast fatigable (FF) MUs the decomposed twitch-like responses frequently have even lower amplitudes than the amplitude of the single twitch. The parameters of the twitch-like responses to individual stimuli appear to depend mainly on the force level reached by a MU when the next stimulus is delivered. This dependence is specific for different MU types [10].

Muscle models composed of MUs [12, 13, 14] give deeper and new insight into the processes of generation and control of the muscle force. In each model, there are many simplifications and assumptions that can lead to errors influencing the muscle model correctness. Summation of equal twitches [15] is the easiest, the most applicable and logical method for modeling tetanic contractions. However, the error made by using this method seems essential, especially for slow MUs [10, 15]. The principal questions are: how do the successive contractions change in comparison to the single twitch? Which parameters determine these changes? How can they be modeled? In the study by Fuglevand et al. (1993) [12] a gain was used for changing the amplitude of successive contractions and this parameter depended on interpulse intervals (IPIs). However, this gain was not specific for the MU type. Moreover, our previous studies have demonstrated that contraction as well as relaxation time parameters of the successive contractions are also variable [10], and that the actual force level is a better predictor of the tetanic force increase than the IPIs [16]. A suitable, physiologically-based approach for prediction of these phenomena, differentiating the three MU types, is still missing.

The aim of the present paper was to find a ***general rule*** for determining the force development for various types of MUs and to develop a new, physiologically-based, mathematical approach for predicting the parameters of twitch-like responses to successive stimuli with different IPIs, delivered in random patterns, which evoke moderately fused tetanic contractions. The derived equations were based on decomposition of tetanic curves of 30 MUs (10 from each physiological type). In order to validate and verify accuracy of the developed mathematical approach we accomplished the following: (1) ***predicted*** the profiles of unfused tetanic contractions for the 30 MUs from the input database used to develop the model; (2) compared the modeled force profiles to the recordings from physiological experiments and to contractions obtained by summation of equal twitches; (3) applied the proposed general mathematical algorithm for prediction of tetanic contractions of three additional MUs outside the database, and not processed during the decomposition and modeling, furthermore evoked at three different patterns of stimuli.

## Methods

### Experimental data

**Fig 1:**
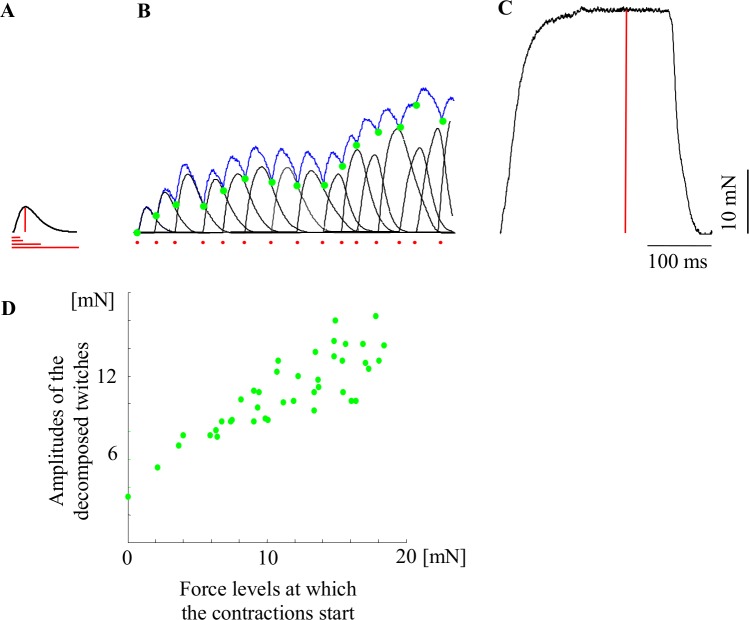
A schematic presentation of the model idea and the necessary input data. A. A single twitch force recording of a MU. The red lines indicate the parameters necessary to model the twitch (see Fig 2 for definitions of these parameters). B. The initial part of an unfused tetanic contraction recording (blue) and models of the successive contractions (black) obtained using a mathematical decomposition of the recording into force responses to individual stimuli. Green dots indicate force levels at the beginnings of the twitch-shape responses to successive stimuli. The red dots indicate the stimuli. C. A recording of a fused tetanic contraction. The red vertical line indicates the maximum tetanus force. D. The amplitudes of the decomposed twitches as a function of the force levels developed by a motor unit at the time moments when the contractions begin. The main aim of the modelling is to predict the course of an unfused tetanic contraction (blue recording) developed during stimulation at variable interpulse intervals using as input data the parameters indicated in red, i.e. the twitch time and force parameters, the maximum tetanus force and the applied pattern of stimuli. The previously observed correlation between the force of the decomposed twitches and the present MUs’ force level gave us a start point for the modelling.

The input data for this modeling study were taken from the results of the decomposition of the recorded curves of unfused tetanic contractions for 30 MUs of the rat medial gastrocnemius muscle–Fig 1. We decomposed 10 MUs of each type: slow (S), fast resistant to fatigue (FR) and fast fatigable (FF). The electrophysiological experiments for the recorded tetanic force curves were presented in our previous paper [11]. The experimental procedures (it was approved by the Local Bioethics Committee in Poznan—permission No 2/2015) of *in vivo* recordings from functionally isolated MUs were there explained in detail. Most importantly, for each MU the following parameters were recorded: the single twitch (Fig 1A), the maximum tetanus at 150 Hz constant rate stimulation (the fused tetanus–Fig 1C) and several unfused tetanic contractions (Fig 1B) at stimulation frequencies evoking tetanus with force levels ranging from 30 to 70% of the maximum tetanus force, and with fusion indices in the range of 0.40 to 0.95. For each frequency the unfused tetanus was evoked by two stimulation patterns composed of 41 pulses. Initially, stimulation at a constant frequency with equal IPIs was applied, and afterwards, stimulation at the same mean frequency, but with non-equal IPIs and within a range of 50–150% of the mean IPI was used [13, 16]. The used MU firing frequencies were: from 10 Hz to 16.6 Hz (mean 12.98 Hz) for S MUs; from 25 Hz to 40 Hz (mean 35.82 Hz) for FR MUs; from 25 Hz to 40 Hz (mean 36.16 Hz) for FF MUs.

### Decomposition of the unfused tetanic curve

Despite the fact that several patterns, which evoked contractions with considerably variable fusion degree, were recorded using different stimulation frequencies, for each of the chosen 30 MUs only one middle-fused tetanic curve with non-equal IPIs was taken for further decomposition and statistics [11]. We used 13 different stimulation patterns of the following frequencies: one of 50 Hz, three of 40 Hz, one of 33.3 Hz, one of 25 Hz, one of 20 Hz, two of 16.6 Hz, two of 14.3 Hz, one of 12.5 Hz and one of 10 Hz (6 patterns are given in S1 File Table B). The experimental curves and the respective IPIs were recorded as text files. These 30 tetanic curves were decomposed using the method described previously [17]. The decomposed 41 successive twitch-like contractions (Fig 1B) were modeled using the 6-parameter analytical function proposed and verified in [9]. The data generalizing all calculated parameters, i.e. the 6 parameters of all decomposed contractions and the maximal force of the fused tetanus, were stored for further analysis.

To verify the accuracy of the decomposition process, a tetanic curve was modeled for each MU, calculating the sum of the models of all decomposed twitch-like contractions according to time distribution of successive pulses in the applied stimulation pattern. The similarity between the recorded and the modeled curves was estimated by using two coefficients proposed and used in [15]. The first one was the fit coefficient, calculated using the formula $FitCo=100\;(1-\sqrt{1-\frac{1}{N}\sum_{i=1}^{N}\Delta_{i}{}^{2}})$, where N is the number of samples and *Δ*_*i*_ is the *i*-th difference between the two curves, normalized to their common maximal value. When the recorded and the modeled curves match perfectly, then *FitCo* = 100%; the lower the *FitCo*, the bigger the difference between them. The other coefficient, *AreaCo*, compares the area under the curves. If *area*_*exp*_ and *area*_*mod*_ are the areas under the recorded and the modeled curves, respectively, $AreaCo=\frac{area_{\exp}}{area_{\mathrm{mod}}}$. If the value of *AreaCo* is 1, the modeled and the recorded forces are similar; if it is higher than 1, the recorded force output of this MU is bigger. If it is less than 1, the model predicts higher total force output during the respective MU activity.

To estimate the error that could be made using a simple algebraic summation of equal twitches (i.e. equal to the model of the first decomposed contraction) using the same stimulation pattern, the same two coefficients were calculated for the experimental and the so modeled curves.

### Approximation of the data obtained from the decomposition of the tetanic curves

**Fig 2:**
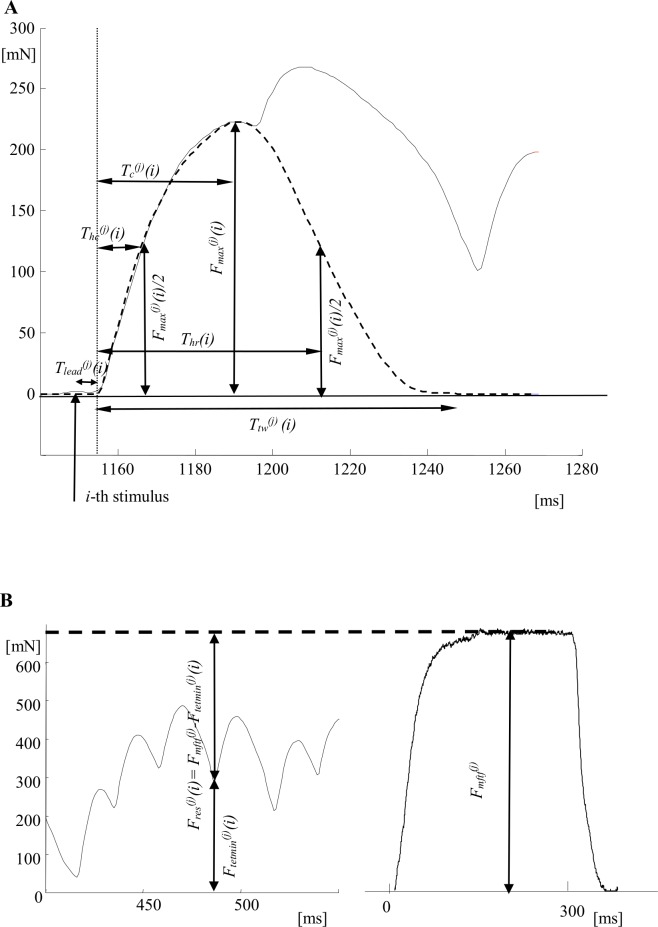
Description of the parameters used for the *j*-th MU recordings. A. The model of the *i*-th decomposed contraction within the unfused tetanus is shown by a dashed line, the black solid line is a piece of the force obtained by subtraction of all previous (*i*-1) contraction models from the experimental tetanic force. The parameters of the *i*-th twitch-like contraction are: *F*_*max*_^*(j)*^*(i)*–the maximum twitch force; *T*_*lead*_^*(j)*^ *(i)*–the lead time, the time between the *i*-th stimulus (its time position is indicated by vertical arrow) and the start of the current *i*-th contraction; *T*_*hc*_^*(j)*^–the half-contraction time, the time from the start of the contraction to the moment when the twitch force reaches a half of its maximal value; *T*_*c*_^*(j)*^–the contraction time, the time from the start of the contraction to the moment when the twitch amplitude reaches it maximal value *F*_*max*_^*(j)*^*(i); T*_*hr*_^*(j)*^*(i)*–the half-relaxation time, the time between the start of the contraction to the moment when during the relaxation, the twitch force decreases to *F*_*max*_^*(j)*^*(i)/*2; *T*_*tw*_^*(j)*^*(i)*—the duration of the current contraction, from the time between the moment when the contraction starts and the moment when the force decreases to 0.01% of *F*_*max*_^*(j)*^*(i)*. The equation describing this bell-shape 6-parameters curve are given in [9]; B. Parameters measured for tetanic contractions presented on a part of the unfused tetanic curve (left) and the maximum fused tetanus (right). *F*_*mftf*_^*(j)*^—the maximal force that a MU develops during stimulation at 150 Hz stimulation frequency (in the fused tetanus). *F*_*tetmin*_^*(j)*^*(i)*—the force level at which the *i*-th contraction starts; *F*_*res*_^*(j)*^*(i) = F*_*mftf*_^*(j)*^*-F*_*tetmin*_^*(j)*^*(i)*—the residual force.

The data from the decomposition of the 30 tetanic curves of the chosen MUs were further used in the approximation procedure. Each decomposed contraction was modeled by the 6-parameter analytical function [9], and the following six parameters were determined for each modeled twitch (Fig 2A): *F*_*max*_^*(j)*^*(i)*, *T*_*lead*_^*(j)*^*(i)*, *T*_*hc*_^*(j)*^*(i)*, *T*_*c*_^*(j)*^*(i)*, *T*_*hr*_^*(j)*^*(i)*, *T*_*tw*_^*(j)*^*(i)*, *i* = 1, 2,…, 41, *j* = 1, 2,…, 30. The index *i* denotes the number of the successive contractions within a tetanus. The index *j* is assigned to different MUs and *j* = 1, 2, …, 10 for slow MUs, *j* = 11, 12,…, 20 for FR MUs, *j* = 21, 22, …, 30 for FF MUs. The meaning of the parameters is as follows: *F*_*max*_^*(j)*^*(i)*—the maximum twitch force; *T*_*lead*_^*(j)*^ *(i)*—the lead time, the time between the *i*-th stimulus and the start of the current *i*-th contraction; *T*_*hc*_^*(j)*^—the half-contraction time, the time from the start of the contraction until the moment when the twitch force reaches one half of its maximal value; *T*_*c*_^*(j)*^—the contraction time, the time from the start of the contraction until the moment when the twitch amplitude reaches its maximal value *F*_*max*_^*(j)*^*(i); T*_*hr*_^*(j)*^*(i)*—the half-relaxation time, the time between the start of the contraction and the moment when during the relaxation, the twitch force decreases to *F*_*max*_^*(j)*^*(i)/*2; *T*_*tw*_^*(j)*^*(i)*—the duration of the current contraction, i.e. the time from the moment when the contraction starts until the moment when the force decreases to 0.01% of *F*_*max*_^*(j)*^*(i)*. The values of these parameters for the first decomposed twitch for the used in this study MUs are given in S1 File Table A. Besides these parameters, for each successive contraction, the level of the force at which it starts, *F*_*tetmin*_^*(j)*^*(i)* (Fig 2B), was also calculated from the force recording. The last parameter, *F*_*mftf*_^*(j)*^, was determined for each MU from the recorded tetanus evoked at 150 Hz as the maximum force of this tetanus and this value was accepted as the maximum tetanic force that a MU is capable of developing. *F*_*res*_^*(j)*^*(i) = F*_*mftf*_^*(j)*^*-F*_*tetmin*_^*(j)*^*(i)* is the force that the MU can still develop at its current state before reaching its maximum force of fused tetanus.

The lead time has a low variability [17], so for modeling purposes it was decided to keep this value unchanged, equal to the value obtained for the first decomposed contraction. Namely, it was accepted that *T*_*lead*_^*(j)*^*(i) = T*_*lead*_^*(j)*^*(1)*.

**Fig 3:**
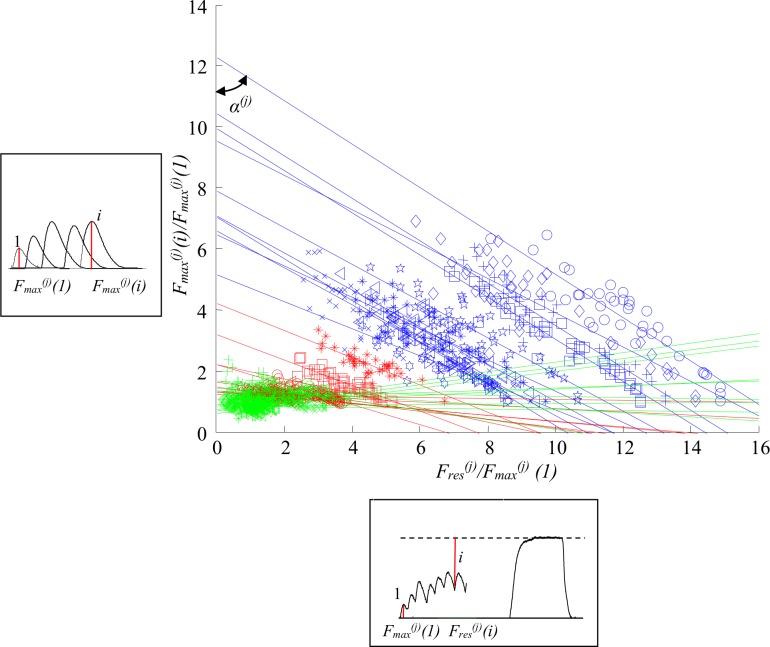
Dependencies between two normalized parameters: *F_max_^(j)^(i)/F_max_^(j)^(1)* and *F_res_^(j)^(i)/F_max_^(j)^(1)*, for all 30 MUs. The two parameters used to calculate data presented on the ordinate are illustrated in a frame left to the axis on an example of a train of decomposed twitches (red lines indicate amplitudes of the first and the *i*-th twitch). Additionally, the parameters used to calculate data presented on the abscissa are illustrated in a frame below the axis on a fragment of the unfused tetanus and the fused tetanus recordings (red lines indicate amplitudes of the first twitch and the residual force for the response to the *i*-th stimulus). The symbols on the main chart marked in blue present the data for slow MUs, in red—the data for FR MUs, and in green—the data for FF MUs. The data for each MU was approximated by straight lines in respective colors: blue for S MUs, red for FR MUs, and green for FF MUs. The angles *α*^*(j)*^ (*j* = 1, 2,…, 30) for each MU were calculated between these lines and the ordinate.

Fig 3 shows the plot of the two parameters, *F*_*max*_^*(j)*^*(i*) and *F*_*res*_^*(j)*^*(i*), normalized to the amplitude of the first decomposed twitch of the respective MU, i.e. *F*_*max*_^*(j)*^*(1*), for all 30 MUs. For each MU a linear approximation of the points (marked with different blue symbols for S MUs, different red symbols for FR MUs and different green symbols for FF MUs) was found, and the angles *α*^*(j)*^ between the approximation lines and the Y axis were calculated. This angle was the smallest for the slow MUs, bigger for the FR MUs, and the largest (even over 90°) for the FF MUs. Values of this angle did not overlap for slow and fast MUs. From physiological point of view, the lines in the Fig 3 mean that for all slow and all FR MUs the amplitude of the next contraction within a tetanus increases when the developed force is closer to the maximal fused tetanic force, and this increase is higher for slow MUs with smaller angle *α*^*(j)*^. The amplitudes, however, have a limit, different for different MUs. This limit was not reached for slow MUs, probably because the tetanic curves were moderately fused and the capacity of slow MUs to develop more force is larger than that of fast MUs. Smaller force amplitude of a contraction for slow MUs is observed when the level of the force at which the contraction starts is far from the maximal possible force for a MU. For some FF MUs, for which *α*^*(j)*^>90^0^, the mentioned force dependencies are opposite–the amplitudes of the contractions increase with the increase of the residual force. The normalized residual force, however, cannot be very high. For the chosen set of FF Mus, the value of the coefficient *F*_*res*_^*(j)*^*(i*)/*F*_*max*_^*(j)*^*(1*) should not exceed 4.

**Fig 4:**
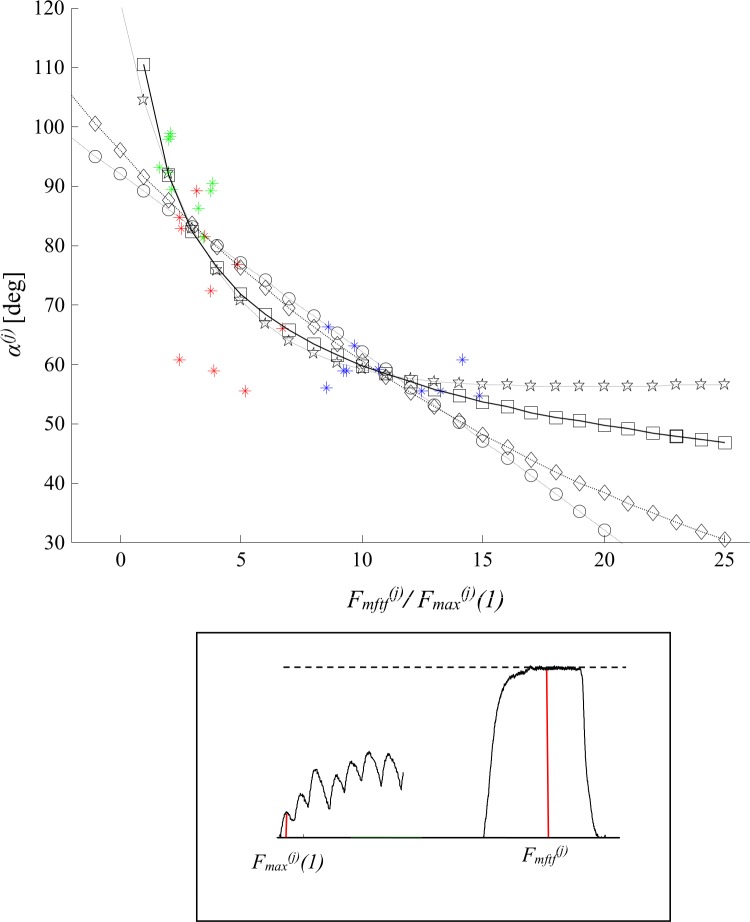
Approximation of the relationships between the angles *α*^*(j)*^ and the parameter *F_mftf_^(j)^* (reflecting the maximum force that the respective MU can develop in the fused tetanus) normalized to the amplitude of the first decomposed contraction. The two parameters used to calculate data presented on the abscissa are illustrated in a frame below the axis on recordings of a fragment of an unfused tetanus and the fused tetanus (red lines indicate amplitudes of the first twitch and the maximum tetanus force). The data for the angles are given in the fourth column of Table 1. S MU—blue asterisks; FR MU—red asterisks; FF—green asterisks. The black dashed curves are different approximations: ‘o’—with a linear model: *y = ax+b*, *a* = -2.994, *b* = 91.96; ‘◊‘—with an exponential model from 1^st^ type: *y = ae*^*bx*^, *a* = 95.8, *b* = -0.04602; ‘✻‘—with an exponential model from 2^nd^ type: *y = ae*^*bx*^*+ce*^*dx*^, *a* = 66.53, *b* = -0.2896, *c* = 54.6, *d* = 0.001297; ‘□’—with a power model (this model is chosen for further modeling and is marked with the bold dashed line): *y = ax*^*b*^, *a* = 108.8, *b* = -0.2603. Here, *y = α1*^*(j*)^ and *x = F*_*mftf*_^*(j)*^*/F*_*max*_^*(j)*^*(1)*.

Fig 3 shows that the calculated angles *α*^*(j)*^ are ***specific*** for each MU, meaning that the development of the tetanic contractions for each MU has its own course. Moreover, clear differences are visible between angles for slow and fast MUs. For these angles different dependencies were checked between different parameters of the decomposed twitches. The relationship between *α*^*(j)*^ and the parameter *F*_*mftf*_^*(j)*^*/F*_*max*_^*(j)*^(1) (Fig 4) was the most perspective for modeling. Moreover, the two parameters, *F*_*mftf*_^*(j)*^ and *F*_*max*_^*(j)*^*(1)*, i.e. the maximum possible MU force and the amplitude of the single twitch, were easily definable and specific for each MU. Several approximations of the above relationship with different linear and non-linear functions were tested using MATLAB functions. The power model shown in Fig 4 with square symbols was determined as the most suitable for our purposes since the calculated root mean square error was the lowest for this particular model. So, the angle *α*^*(j)*^ was calculated by the equation:

$$\alpha^{\left(j\right)}=108.8{\left(F_{mftf}{}^{\left(j\right)}/F_{max}{}^{\left(j\right)}(1)\right)}^{\left(-0.2603\right)}.$$ (1)

Having the specific angle *α*^*(j)*^ for a MU, and constituting the equations of the straight lines shown in Fig 3, the amplitude of each *i*-th contraction within a tetanic curve could be calculated for each *j*-th MU by the following equation:

$$F_{max}{}^{\left(j\right)}(i)=(1+cot(\alpha^{\left(j\right)})F_{tet\;min}{}^{\left(j\right)}(i)/F_{max}{}^{\left(j\right)}(1))F_{max}{}^{\left(j\right)}(1).$$ (2)

**Fig 5:**
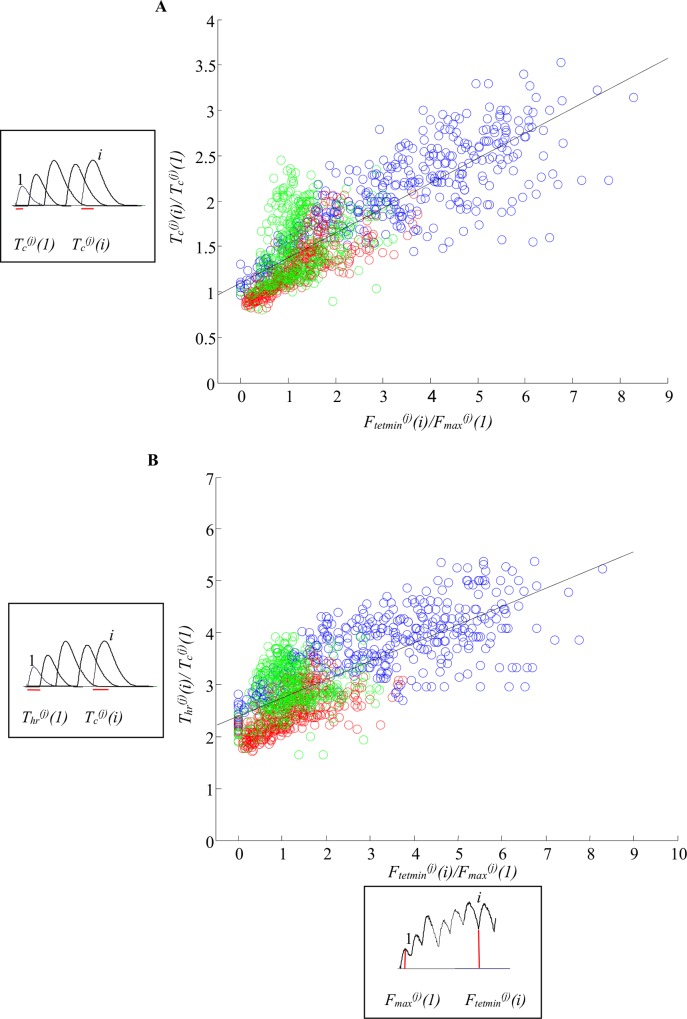
Linear approximation of the data for the contraction and the half-relaxation times. A. The dependence between the contraction time *T*_*c*_^*(j)*^*(i)* and the parameter *F*_*tetmin*_^*(j)*^ (reflecting the MU force level at which the next contraction starts), normalized according to the parameters of the first decomposed twitch-like contraction *T*_*c*_^*(j*)^(1) and *F*_*max*_^*(j)*^(1), respectively. The equation of the straight line is: *y = p*_*1*_*+p*_*2*_*x*, and *p*_*1*_ = 1.104, *p*_*2*_ = 0.274; B. The dependence between the half-relaxation time *T*_*hr*_^*(j)*^*(i)* and the parameter *F*_*tetmin*_^*(j)*^*(i)*, normalized to the parameters of the first decomposed twitch-like contraction *T*_*hr*_^*(j*)^(1) and *F*_*max*_^*(j)*^(1), respectively. The equation of the straight line is: *y = p*_*1*_*+p*_*2*_*x*, and *p*_*1*_ = 2.397, *p*_*2*_ = 0.3509; blue circles—S MUs; red circles—FR MUs; green circles—FF MUs. The two twitch time parameters used to calculate the data presented on the ordinate are illustrated in frames left to the axis on the example of a series of decomposed twitches (red lines indicate the contraction time for the first and the *i*-th twitch in A, and the half-relaxation time for the first and for the *i*-th twitch in B). Additionally, the parameters used to calculate data presented on the abscissa are illustrated in a frame below the axis on a fragment of the unfused tetanus recording (red lines indicate amplitudes of the first twitch and the force level at which the *i*-th contraction starts).

The next two parameters of the successive twitch-like responses—contraction and half-relaxation times—were hypothesized to depend on the actual MU force level at which the *i*-th contraction started, i.e. *F*_*tetmin*_^*(j)*^*(i)* (Fig 2B). The plots in Fig 5 suggest an approximation of the points with linear functions. Thus, the following equations for calculation of these two parameters could be written:

$$T_{c}^{\left(j\right)}(i)=(1.04+0.274\;F_{tet\;min}{}^{\left(j\right)}(i)/F_{max}{}^{\left(j\right)}(1))T_{c}^{\left(j\right)}(1),$$ (3)

$$T_{hr}{}^{\left(j\right)}(i)=(2.397+0.3509\;F_{tet\;min}{}^{\left(j\right)}(i)/F_{max}{}^{\left(j\right)}(1))T_{c}^{\left(j\right)}(1).$$ (4)

The choice of the linear models shown in Fig 5 was confirmed by the high correlation coefficients, which were 0.8059 for *T*_*c*_ and 0.7314 for *T*_*hr*_.

The remaining two parameters, i.e. the half-contraction time and the duration of the twitch, were supposed to be changed proportionally to the contraction and the half-relaxation times, respectively. Hence, the following equations were used for these two parameters:

$$T_{hc}{}^{\left(j\right)}(i)=T_{hc}{}^{\left(j\right)}(1)(T_{c}^{\left(j\right)}(i)/T_{c}^{\left(j\right)}(1)),$$ (5)

$$T_{tw}{}^{\left(j\right)}(i)=T_{tw}{}^{\left(j\right)}(1)(T_{hr}{}^{\left(j\right)}(i)/T_{hr}{}^{\left(j\right)}(1)).$$ (6)

### The tetanus force modeling procedure

The Eqs (2), (3) and (4) include as a predictor the force level at which each contraction starts, i.e. *F*_*tetmin*_^*(j)*^*(i)*. This is why first, the input parameters for modeling an unfused tetanic force curve for the *j*-th MU were specific for each MU, i.e. the force parameters *F*_*mftf*_^*(j)*^ and *F*_*max*_^*(j*)^(1) and the twitch time parameters *T*_*lead*_^*(j)*^*(*1), *T*_*c*_^*(j)*^*(*1), *T*_*hr*_^*(j)*^*(*1), which could be estimated from the first decomposed contraction or from the recorded individual MU twitch, or were specific for the applied stimulation pattern, i.e. *F*_*tetmin*_^*(j)*^*(i)* (Fig 2). The latter parameter was at first calculated as local minima of the recorded tetanic curves. Having these parameters, the angle *α*^*(j)*^ was calculated using the Eq (1). Then, the remaining parameters of the models of the successive contractions were calculated using Eqs (2–6). Note that the lead times were constants for all contractions of each MU. The twitches were modeled using the 6-parameter analytical function [9] and were summated according to the actual stimulation pattern. The obtained modeled force curve was compared with the respective experimental recording, and the two coefficients for estimation of the similarity between curves were calculated (Table 1). The high accuracy of the modeling was demonstrated by the values of the two fit coefficients (Table 1, *FitCo2* and *AreaCo2*).

**Table 1:** Calculated angles and coefficients reflecting the similarity between the experimental curves and the predicted curves for all 33 MUs. *FitCo1* and *AreaCo1* are these coefficients when the modeled curve is obtained as a sum of equal to the model of the first contraction twitches, according to the respective stimulation pattern. *α1*^*(j)*^ is the angle calculated by using the Eq (1). *FitCo2* and *AreaCo2* are the coefficients for the experimental and modeled curve (the calculated experimental values of *F*_*tetmin*_^*(j)*^*(i)* are used as input parameters for the prediction). *FitCo3* and *AreaCo3* are the coefficients for the experimental and modeled curve using the same angles *α1*^*(j)*^ but for full prediction algorithm (i.e. values of F_tetmin_^(j)^(i) are also predicted consecutively). *α2*^*(j)*^ is the angle obtained by a sensitivity analysis so that the modeled curve is the most similar to the experimental one, and *FitCo4* and *AreaCo4* are the respective coefficients. *α3*^*(j)*^ is the improved angle using the Eq (11) (see Fig 8), and *FitCo5* and *AreaCo5* are the respective coefficients.

*MU*	*FitCo1*	*AreaCo1*	*α1*^*(j)*^	*FitCo2*	*AreaCo2*	*FitCo3*	*AreaCo3*	*α2*^*(j)*^	*FitCo4*	*AreaCo4*	*α3*^*(j)*^	*FitCo5*	*AreaCo5*
S1	57.6964	6.2793	53.8892	88.5207	1.3240	65.1148	3.3396	44.5	85.7297	1.2417	50.1630	69.5218	2.1219
S2	44.3594	5.8069	60.2293	89.7490	1.1761	56.4147	2.8651	53.2	86.9483	0.9978	57.3759	62.6939	2.0525
S3	44.8564	6.6772	55.5446	88.1151	1.2042	52.3472	3.6521	45.1	83.6021	1.2411	52.0301	59.2353	2.8785
S4	38.9658	5.6567	60.9302	90.2219	0.9382	74.0258	1.5313	60.1	78.5348	1.2536	60.2272	74.4744	1.5147
S5	60.5076	4.4310	56.3657	92.0639	1.2080	70.9010	2.3328	48.1	85.4634	1.4254	52.9605	73.8448	2.0644
S6	45.8927	7.3539	54.5858	91.9735	1.0214	56.2810	3.2983	50.9	81.2577	1.2501	50.9472	78.2303	1.5063
S7	64.0833	3.3468	62.2282	85.5089	1.4022	72.4592	2.2058	50.1	85.5148	1.3642	59.6837	73.8274	2.0754
S8	54.1593	4.2047	60.6930	92.0619	1.1467	71.1615	1.9452	57.4	89.4606	1.0966	57.9099	83.8329	1.3639
S9	54.7910	4.8920	58.7387	93.2627	1.1043	76.2017	1.7479	57.0	89.1927	1.1630	55.6652	89.2803	1.1321
S10	60.4031	3.5424	62.1363	88.1446	1.3001	72.5132	1.9934	53.0	87.1135	1.1861	59.5773	75.0600	1.8275
FR1	88.0511	1.1391	76.8619	92.3322	0.8706	84.6699	0.7338	87.0	91.6580	0.9528	77.0271	90.9951	0.8929
FR2	59.9559	2.9344	66.2353	92.1104	0.8830	53.8544	0.0365	74.1	90.6971	1.0359	74.3563	88.7851	0.9122
FR3	83.3022	1.3677	72.1234	93.0732	0.8788	84.7762	0.8788	82.0	92.1265	0.9227	79.3288	91.2859	0.8656
FR4	81.4146	1.3834	76.2942	95.4785	0.9569	91.3040	0.8539	81.0	94.3569	0.9883	76.3405	91.3591	0.8552
FR5	75.3674	1.7145	70.9255	92.2023	0.8806	75.5227	0.5635	79.0	91.9652	0.9673	79.9003	91.8007	1.0027
FR6	88.8640	1.1649	85.7127	91.5697	0.8660	86.6490	0.7793	92.0	91.6128	0.9209	87.8649	90.4403	0.8937
FR7	84.2008	1.3129	85.3680	94.2266	0.9202	90.1883	0.8436	89.1	93.1131	0.9303	87.4382	92.1057	0.8935
FR8	75.7108	1.5911	78.3537	94.6322	0.9903	92.3706	0.9116	80.0	92.7829	0.9586	86.8365	92.5900	0.9256
FR9	81.7899	1.3221	85.9399	93.383	1.0278	92.5908	1.0046	85.6	92.6213	0.9960	88.1462	92.6213	0.9960
FR10	73.6190	1.6691	80.2466	95.0035	0.9975	93.7894	0.9430	80.5	94.0344	0.9521	81.1426	94.5252	0.9751
FF1	72.4075	1.9102	76.9690	94.9658	0.9724	91.1809	0.8528	80.1	94.4998	0.9626	77.1567	91.4560	0.8599
FF2	76.7497	1.4399	90.5304	90.8634	1.0797	91.0772	1.0823	90.0	91.4555	1.0678	90.8644	90.8076	1.0914
FF3	67.3458	1.9464	78.6948	90.4582	0.8871	80.5306	0.6936	84.0	94.1159	1.0386	83.2512	93.9650	0.9951
FF4	84.1144	1.2901	76.6350	84.6073	0.7493	73.9777	0.5352	96.0	92.4101	1.0874	91.7525	94.1122	0.9788
FF5	72.2486	1.7899	80.0172	95.5517	0.9791	93.0477	0.9057	82.1	95.8175	0.9946	80.8626	94.5983	0.9443
FF6	79.0362	1.3836	95.8677	86.2715	1.2142	86.2816	1.2169	91.0	90.5627	1.1236	100.5887	90.5636	1.1241
FF7	81.6039	1.3208	89.4323	92.4764	0.9110	91.2557	0.8803	95.0	93.7330	0.9963	92.4909	93.1696	0.9445
FF8	89.4961	0.9111	90.4512	89.9263	0.9633	89.8191	0.9013	96.0	92.0044	0.9910	93.7652	91.4594	0.9555
FF9	88.2175	1.1828	89.5973	93.4030	1.0443	93.7385	1.0333	88.7	93.8819	1.0120	92.6970	93.7100	1.0357
FF10	88.4189	1.0587	89.1032	90.4339	0.9852	90.6219	0.9825	91.0	90.7065	1.0115	92.0799	90.5926	1.0279
S11	64.6536	2.2146	71.9749	92.7455	1.0338	91.1586	0.9669	73.0	91.5871	1.0245	72.1514	91.3145	0.9771
FR11	83.3022	1.3677	72.1234	93.0732	1.3677	84.7762	0.6943	83.1	92.3100	0.9435	81.7053	92.0441	0.9086
FF11	91.1095	1.0746	89.6039	92.0953	1.0746	92.4251	1.0622	87.0	92.9321	1.0120	92.7053	91.9152	1.0833

However, the weakness of this modeling approach was the necessity of data concerning the actual force level, i.e. *F*_*tetmin*_^*(j)*^*(i)*. For modeling purposes, this is a big obstacle when experimental data is missing for a given MU. That is why this parameter was also calculated in the next modeling step.

**Fig 6:**
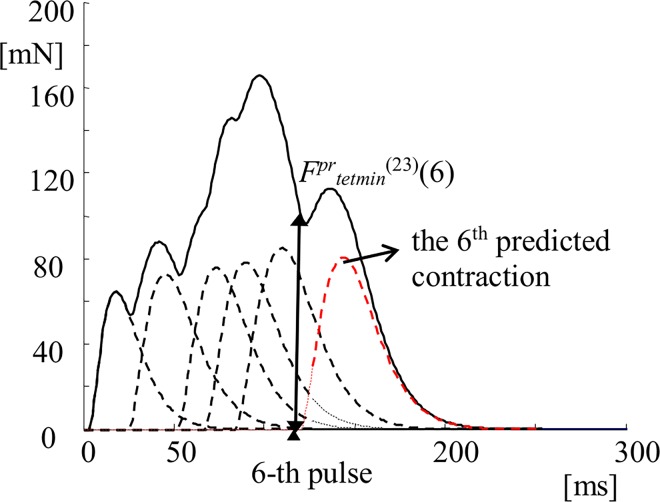
Illustration of the calculation of the parameter *F^pr^_tetmin_^(j)^*(*i*) for the 23^th^ MU after adding the models of all preceding contractions. The first five models (plotted by using black dashed lines) are summed and the accumulated force is plotted as a solid black line; the value of the force *F*^*pr*^_*tetmin*_^*(23*)^ (6) is computed at the moment when the 6^th^ pulse comes and the next, 6^th^ contraction, is calculated using this value. The model of this 6^th^ contraction is red dashed line. The solid black line after the appearance of the 6-th pulse shows the addition of the force evoked by the 6^th^ pulse to the previous five contractions.

Instead of taking the parameters *F*_*tetmin*_^*(j)*^*(i)* from the recorded tetanic curves, *F*_*tetmin*_^*(j)*^*(i)* can also be predicted using the preceding modeled twitches (Fig 6). If the *i*-th twitch-like contraction of the *j*-th MU has the following force as function of time *t* (for *t* [0, *t*_*exp*_], where *t*_*exp*_ is the time duration of the experimental curve):

$$F_{tw}{}^{\left(j\right)}(i)(t)=f(t,F_{max}{}^{\left(j\right)}(i),T_{lead}{}^{\left(j\right)}(i),T_{hc}{}^{\left(j\right)}(i),T_{c}^{\left(j\right)}(i),T_{hr}{}^{\left(j\right)}(i),T_{tw}{}^{\left(j\right)}(i)),$$ (7)

then the force level at which the *n*-th pulse comes, *F*^*pr*^_*tetmin*_^*(j)*^*(n)*, will be:

$$F^{pr}{{}_{tet\;min}}^{\left(j\right)}(n)={\sum_{i=1}^{n-1}F_{tw}}^{\left(j\right)}(i)(t_{IMP(n)}),$$ (8)

where *t*_*IMP(n)*_ is the time moment of the *n*-th pulse (*n* = 1, 2,…, 41) in the pattern used for the *j*-th MU. This mathematical process is visualized in Fig 6 where the first five contraction models are summed, the force developed by the MU at the moment of the 6-th pulse, *F*^*pr*^_*tetmin*_ is calculated and used in Eqs (2), (3) and (4) as a predictor in order to predict the 6^th^ contraction.

For this type of more complex force prediction, the so called “full prediction”, the procedure is the following: the *α* angle is calculated by the Eq (1), then the first contraction is modeled having *F*_*tetmin*_^*(j)*^*(1) = 0;* the force level *F*^*pr*^_*tetmin*_^*(j*)^(2) is computed from the first contraction as the force level at the moment when the second pulse is delivered, and this value is used instead of *F*_*tetmin*_^*(j*)^(2) in the Eqs (2–4). Hence, the second twitch model is generated. The first and the second models are added and the force level at which the third pulse comes is calculated. This is the value *F*^*pr*^_*tetmin*_^*(j*)^(3), which is used instead of *F*_*tetmin*_^*(j*)^(3) in the Eqs (2–4). This procedure continues until the last, 41^st^ pulse. **This way a tetanic force can be predicted from 6 available parameters of the single twitch, the maximal fused tetanus force and a given stimulation pattern.**

## Results

The curves obtained by summation of the models of all 41 decomposed contractions well resembled the recorded tetanic curves for all 30 MUs. The mean value of the fit coefficients between the recorded and the modeled curves was 98.6829 (range 98.0604–99.2647). The mean value of the *AreaCo* was 1.0068 (range 1.0003–1.0154), i.e. the modeling error was below 2%. There were no differences in the preciseness of the modeling between different types of MUs. This confirmed the preciseness of the decomposition of all 30 tetanic curves and completeness of the teaching database.

**Fig 7:**
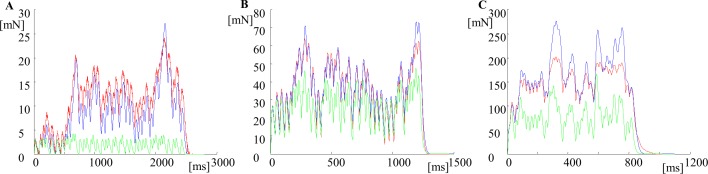
The effect of the application of the new approach for prediction of the successive contraction for three MUs. Comparison between the recorded tetanic curves (red), the force curves obtained by summation of equal twitches according to the same stimulation pattern (green), and the curves predicted by the approximation approach (blue) using the angles α1^(i)^ from Table 1. A. A slow MU (S1 in Table 1), the stimulation pattern with interpulse intervals IPI1 is used; B. A FR MU (FR4 in Table 1), the stimulation pattern with interpulse intervals IPI2 is used; C. A FF MU (FF2 in Table 1), the stimulation pattern with interpulse intervals IPI3 is used. IPIs are given in S1 File Table B. Note that the time and the force scales are different for the three MUs.

When using all input parameters (S1 File Table A) for the generation of the prediction model curves, including the force level at which each successive contraction starts taken from the force recordings, the tetanic curves modeled by using the approximation approach, i.e. applying the Eqs 1–6, were much closer to the recorded ones than to the curves obtained by summing equal twitches for all MUs (Fig 7). Note that the IPIs for the stimulation patterns used for these three tetanic curves are given in S1 File Table B. The visual observations from these three plots were confirmed by the values of the two coefficients given in Table 1. The number 1 was assigned to the coefficients estimating the similarity between the recorded curve and the curve obtained by summation of equal twitches. The number 2 was assigned to the coefficients, estimating the similarity between the recorded curve and the curve obtained by the summation of the contraction models, whose parameters were predicted by the approximation. *FitCo1* was always lower than *FitCo2* while *AreaCo1* was always bigger than *AreaCo2* (with the exception of FF8), and AreaCo1 was usually closer to 1. The most apparent differences were observed for slow MUs. For slow MUs the difference between the two fit coefficients, *FitCo1-FitCo2*, varied between 21.4256 (for S7) and 51.2561 (for S4, this was also the maximum value for all MUs). Smaller values for *FitCo1-FitCo2* were observed for FF MUs; the minimum value was 0.4302 (for FF8), and the maximum value was 23.1124 (for FF3). The minimum absolute value of *AreaCo1-AreaCo2* was 0.0522 (for FF8), while the maximum was 6.3325 (for S6). To summarize, the force curve predicted by the model using the new proposed approach was always considerably closer to the recorded tetanic force than the one obtained by summation of equal twitches. This conclusion was more evident for the slow MUs.

Table 1 and Fig 3 show the range of the angle α^(j)^, which varied from 53.8892° to 95.8677°, and increased from slow MUs to fast ones. For three FF MUs, this angle was even bigger than 90°. When this angle was equal to 90° the approximation line for a particular MU was horizontal and all successive contractions had one and the same force amplitude, independently of the stimulation pattern. These twitch-like contractions only differed in their shapes by a prolonged duration, because of the dependencies shown in Fig 5 and the Eqs (3–6). Within the population of the 30 MUs taken as input data, the relationship between the maximal possible tetanus force of a MU and the amplitude of its single twitch force (hence, *F*_*mftf*_^*(j)*^/*F*_*max*_^*(j)*^(1)—see S1 File Table A), was from 1.6260 (for FF6) to 14.8666 (for S1), and this relationship explicitly determines the angle α^(j)^ (see Eq (1)) for a particular MU.

In the majority of FF MUs, some values of *F*_*max*_^*(j)*^*(i)/F*_*max*_^*(j*)^(1) were below 1 (Fig 3). This means that some decomposed contractions had amplitudes lower than the first contraction. This never occurred for slow and FR MUs.

The plots in Fig 3 depend on two important parameters, specific for each MU—the maximal possible tetanus force that a MU can develop and the amplitude of the first decomposed twitch. Both are subjects of error during modeling. This especially refers to changes of the parameters of the twitches due to physiological processes, such as a potentiation at the beginning of an experiment or fatigue at the end. As an effect, the recorded force can vary within the experiment. On the other hand, the maximal tetanic force can decrease progressively during an experiment due to the development of fatigue, especially in FF MUs.

To check whether the proposed mathematical approach could be applied for various MUs in rat medial gastrocnemius (i.e. MUs not included in the initial database) ***three additional*** MUs, one of each type (S11, FR11 and FF11 in Table 1 and in S1 File Table A), previously not taken for decomposition and modeling procedures, were chosen for verification. The recorded tetanic curves of these MUs had similar fusion indices, but stimulation patterns (given in S1 File Table B) were different from all those previously used. The predicted force curves closely resembled the recorded curves for S11, FR11, and FF11 MUs. This was confirmed by the coefficients *FitCo2 and AreaCo2* given in Table 1.

**Fig 8:**
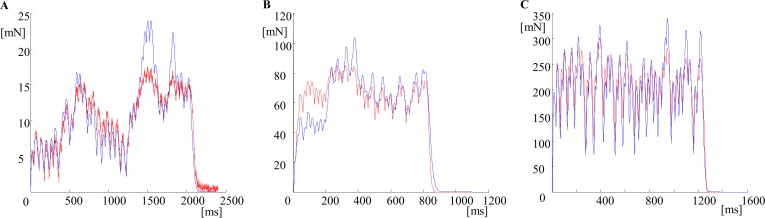
Comparison between experimental tetani and the force curves, obtained through the new approximation approach. Full prediction of three additional tetanic curves, obtained by stimulation with three new patterns, applied to three MUs not included in the input database. Red color—the recorded curve; blue color—the predicted force curve. A. A slow MU stimulated with the mean frequency of 20 Hz (S11 in Table 1), the stimulation pattern with interpulse intervals IPI4 is used; B. A FR MU stimulated with the mean frequency of 50 Hz (FR11 in Table 1), the stimulation pattern with interpulse intervals IPI5 is used; C. A FF MU stimulated with the mean frequency of 33.3 Hz (FF11 in Table 1), the stimulation pattern with interpulse interval IPI6 is used. The values of the angles *α1*^*(i)*^ are shown in Table 1. IPIs are given in S1 File Table B. Note that the time and the force scales are different for the three MUs.

The next step in the modeling was an attempt to fully predict the course of a MU tetanic contraction, calculating the consecutive force levels at which the subsequent pulses were delivered, using Eq (8), but not taking them from the recorded tetani. Hence, only the parameters specific for a given MU and the stimulation pattern would be necessary for a tetanic force prediction. This so called “full prediction” was made for all 33 MUs. *FitCo3* and *AreaCo3* in Table 1 reflect the similarity between the recorded force curve and the one modeled by the full prediction algorithm. The recorded and the predicted curves for the S11, FR11 and FF11 MUs, which are outside the database, are shown in Fig 8.

Comparing *FitCo3* and *AreaCo3* with *FitCo2* and *AreaCo2* from Table 1 it can be concluded that the preciseness of the prediction changes for the worse since additional inaccuracies were put into the calculations—the force level at which the next contractions start. Possible mistakes could be also due to the calculation of the angle *α*^*(j)*^ from Eq (1), since this parameter considerably influences the force amplitude of the consecutive contractions. Small changes in this angle could potentialy increase or decrease the tetanic force. Moreover, the dependence shown in Fig 4 did not appear to be sufficiently precise. For these reasons, a sensitivity analysis was performed. For each MU from Table 1, the angle *α1*^*(j)*^, calculated using Eq (1), was changed within a suitable limit, and a value giving the best prediction was chosen as the angle *α2*^*(j)*^. The limits for the sensitivity analysis were the integer value of *α1*^*(j)*^ plus/minus 20° with steps of 0.1°. The best prediction was chosen on a basis of both coefficients *FitCo4* and *AreaCo4* ***–***they had to be as close as possible to 100% and 1, respectively. Table 1 shows values of ***α*** angle corresponding to the most accurate prediction of the tetanic curves. For all slow MUs (except S11) this angle was decreased, and for one MU it was decreased even by 12°. For nearly all fast MU (except for FR9, FF2, FF6, FF9 and FF11) this angle was increased, and for FF4 it was increased even by 19°.

**Fig 9:**
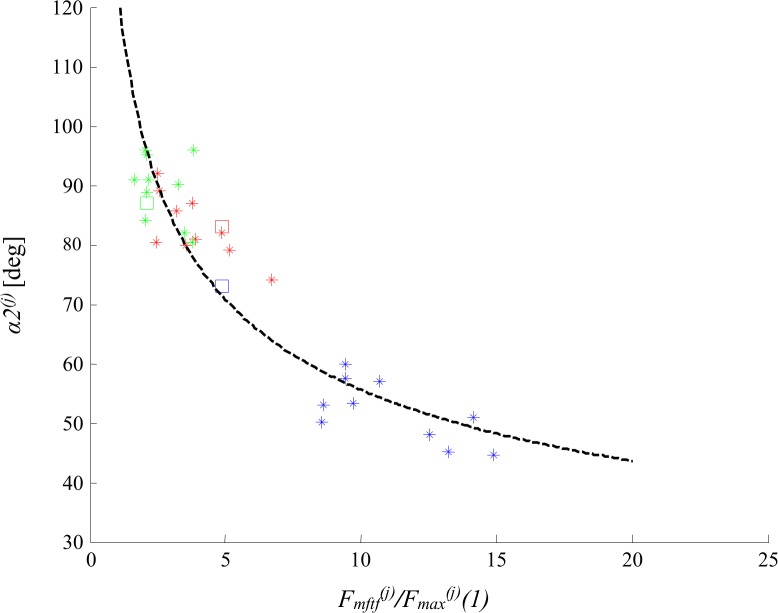
Approximation of the relationships between the *improved* angles, i.e. *α*2^*(j)*^ in Table 1, and the parameter *F_mftf_^(j)^* normalized to the amplitude of the first decomposed contraction of a MU. S MUs—blue asterisks; FR MUs—red asterisks; FF MUs—green asterisks. The squares present the three additional MUs (S11, FR11 and FF11). The black dashed curve is the new approximation with a power model *y = ax*^*b*^, where a = 117.2, b = -0.3144, *y = α2* ^*(j)*^ and *x = F*_*mftf*_^*(j)*^*/F*_*max*_^*(j)*^*(1)*.

These observations were used to improve the Eq (1). Fig 9, where these angles *α*^*(j)*^, obtained after the sensitivity analysis, are plotted versus *F*_*mftf*_^*(j)*^*/F*_*max*_^*(j*)^, shows a better fit function. An ameliorated equation for the dependence in Fig 9 was obtained, namely:

$$\alpha^{\left(j\right)}=117.2{\left(F_{mftf}{}^{\left(j\right)}/F_{max}{}^{\left(j\right)}(1)\right)}^{\left(-0.3144\right)}.$$ (9)

The full prediction was performed again for all 33 MUs, using the new equation. The final values of the angle were calculated using the Eq (9), i.e. *α3*^*(j)*^ in Table 1. The coefficients *FitCo5* and *AreaCo5* are in general better than *FitCo3* and *AreaCo3*, although there are not as good as *FitCo2* and *AreaCo2*. This allows us to suppose that the precise values of *F*_*tetmin*_^*(j)*^*(i)* are very important for predicting the successive contractions and that a further sensitivity analysis with respect to the contraction and the half-relaxation times is necessary.

## Discussion

This paper has two achievements. First, the proposed approach can be used to develop more realistic muscle models composed of a set of MUs, which may be used to study processes of control of the skeletal muscle force. Second, the differences in the force development during repetitive activation of MUs from various types have become clearer, and the separation between the three types of MUs can now be made based on the new observations, namely the values of the angle *α*^*(j)*^ (Figs 3 and 9). The application of the angle *α* is the major novelty of this muscle model. It appears to be a parameter specific for respective types of MUs and enables us to predict the course of unfused tetanic contractions using the same equations and MU quantities for arbitrary stimulation patterns with variable interpulse intervals. Fig 3 shows that each MU has its own specific angle. The values for *α1*^*(j)*^ for slow MUs ranged from 53.8892° to 62.2282° (S11 seems an exception), and for fast units—from 66.2353° to 95.8677°, so these ranges were not overlapping (Table 1). The values of the angle *α1*^*(j)*^ overlapped for FF and FR MUs, but on average, were higher for FF units. The same conclusions can be made for *α2*^*(j)*^ and *α3*^*(j)*^. For slow MUs, *α2*^*(j)*^ is between 44.5° and 60.1°, and for fast MUs, *α2*^*(j)*^ is between 74° and 96°. For slow MUs, *α3*^*(j)*^ is between 50.1630° and 60.2272°, and for fast MUs, *α3*^*(j)*^ is between 74.3563° and 100.5887°. In general, this angle determines whether the amplitudes of the successive contractions within an unfused tetanus would increase (*α*^*(j)*^*<*90°), remain constant (*α*^*(j)*^ = 90°) or decrease (*α*^*(j)*^>90°) during tetanic force development. The range of the values of this angle depends on the relationship *F*_*mftf*_^*(j)*^*/F*_*max*_^*(j*)^(1), so one may expect that for another population of MUs (other muscles, muscles in various species), ranges that are different from those presented in Table 1 would be obtained.

The MU force varies within a range from the single twitch amplitude up to the maximum (fused) tetanus force. This range is usually expressed by a twitch-to-tetanus force ratio, and this ratio is lowest for S MUs and highest for FF units. For the rat medial gastrocnemius, the mean values of this ratio amount to 0.13±0.05, 0.19±0.06 and 0.28±0.08 for S, FR and FF MUs, respectively [18]. Accepting that the minimal value of the twitch-to-tetanus ratio is about 0.05, and that *p* denotes this ratio, the inequality 0.05*≤p≤*1 can be written since the twitch amplitude has to be lower than the maximal possible MU force. This can be rewritten as 0.05≤*F*_*max*_^*(j*)^(1)/*F*_*mftf*_^*(j)*^≤1. Then because of the improved Eq (9),

$$\alpha^{\left(j\right)}=117.2{\left(1/p\right)}^{\left(-0.3144\right)}.$$ (10)

Hence, the maximal value of the angle is 117.2^0^ and the minimal one is 45.6966^0^. So, the Eq (9) has clear physiological basis.

Neither Eq (1) nor Eq (9) imposed a mathematical limit for the angle. However, since an MU force may vary from a minimum activity during a single twitch up to the maximum force in the fused tetanic contraction, a limit based on the maximum possible tetanic force should be taken into consideration during modeling more fused tetani. From a physiological point of view, the power model lines in Fig 4 and Fig 9 have to be limited by vertical lines crossing 1 at the abscissa, since the amplitudes of the individual twitches cannot exceed the maximal tetanus force.

Nevertheless, the proposed equations predict some limits in the force development. They have physiological interpretation, too. For example the Eq (2) can be rewritten in another form:

$$\frac{F_{max}{}^{\left(j\right)}(i)}{F_{max}{}^{\left(j\right)}(1)}=1+\cot(\alpha^{\left(j\right)})\frac{F_{tetmin}{}^{\left(j\right)}(i)}{F_{max}{}^{\left(j\right)}(1)}.$$ (11)

**Fig 10:**
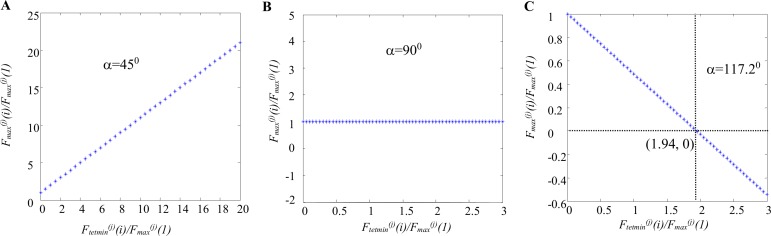
Relationship between the two parameters, *F_tetmin_^(j)^(i)* and *F_max_^(j)^(i)*, both normalized according to the first twitch amplitude, presented for three border values of the angle *α*. The Eq (11) is used for calculations. This dependence is linear and is shown by blue asterisks. If *F*_*tetmin*_^*(j)*^*(i)* = 0, i.e. the contraction starts from a fully relaxed MU, the amplitude of the evoked contraction will be equal to the amplitude of the individual twitch. A. *α =* 450 –the amplitudes of the successive contractions increase when the levels of the force at which a contraction starts increase, and *F*_*max*_^*(j)*^*(i)* are always bigger than *F*_*max*_^*(j*)^(1*)*. B. *α =* 900 –the successive contractions always have amplitudes equal to the maximal force of the single twitch. C. *α =* 117.20 –the amplitudes of the successive contractions decrease when *F*_*tetmin*_^*(j)*^*(i)* increase. Since *F*_*max*_^*(j)*^*(i)*≥0 the normalized value of *F*_*tetmin*_^*(j)*^*(i*) cannot exceed the value of 1.94 (the crossed point of the dotted vertical and horizontal lines). When *F*_*tetmin*_^*(j)*^*(i)/ F*_*max*_^*(j)*^*(1)* = 1.94, a MU is unable to respond with a new contraction.

When *F*_*tetmin*_^*(j)*^*(i)* = 0, i.e. when the contraction starts from fully relaxed MU, the developed contraction would have nearly identical amplitude with the amplitude of the first (single) twitch. If *α*^*(j*)^<90° and if *F*_*tetmin*_^*(j)*^*(i)>0*, i.e. in case when the next pulse comes before the full relaxation of a MU, then the amplitudes of the next contractions would always be bigger than *F*_*max*_^*(j*)^(1), since cot(*α*^*(j*)^)>0. If *α*^*(j*)^ = 90°, the amplitudes of the next contractions would always be the same as *F*_*max*_^*(j*)^(1). The third case, when *α*^*(j*)^>90°, seems especially interesting, since the amplitudes would decrease. Three examples are given in Fig 10 for the three border variants of the angle: *α*^*(j*)^ = 45^0^, *α*^*(j*)^ = 90^0^ and *α*^*(j*)^ = 117.2°. As can be concluded from Fig 3 and Table 1, for all slow and FR MUs, *α*^*(j*)^<90^0^. In such a case (Fig 10A) the amplitude of a contraction within a tetanus increases when the level at which the contraction starts is higher. If *F*_*tetmin*_^*(j)*^*(i)* = 0, i.e. in the case when a MU is fully relaxed, the contraction will have the same amplitude as the single twitch (the first point in Fig 10A). Upper limit of *F*_*max*_^*(j)*^*(i*) and *F*_*tetmin*_^*(j)*^*(i)* has to be imposed further by inequality constraints for more fused tetani. When *α*^*(j*)^ = 90^0^ (Fig 10B) the amplitudes of all contractions of this MU will be equal, i.e. *F*_*max*_^*(j)*^*(i*) = *F*_*max*_^*(j)*^*(1*). When *α*^*(j*)^ >90^0^, which can happen for some FF MU, a decrease of all amplitudes with respect to *F*_*max*_^*(j*)^(1) is visible. From the inequality *F*_*max*_^*(j*)^(i)≥0, a border value for *F*_*tetmin*_^*(j)*^*(i)* (see point (0, 1.94)) can be obtained which value, however, is different for different MUs with different angles. Namely, the amplitude of subsequent contractions cannot exceed 1.94 times the force level at which the contraction starts for this particular border variant, i.e. for this particular fast MU. If the level of a MU force exceeds 1.94 times *F*_*max*_^*(j)*^*(1)* when the next stimulus is delivered, the MU will be not able to respond with a new contraction, i.e. the MU will not generate more force.

The MU force can be regulated by changes in the motoneuronal firing rate, and during voluntary activity, motoneurons generate trains of pulses at variable intervals, which substantially influence the development and profiles of tetanic forces [19–21]. In our study, 13 different patterns with different mean frequencies were used for stimulation of the MUs. This diversity of the stimulation patterns was applied in the experiments to ensure that statistical data is reliable for approximation (S1 File Table B). Moreover, the approach was validated using the tetanic force curves obtained by stimulations with three additional patterns, different from those used previously. It has to be mentioned also that the range of the contractile parameters of the 33 investigated MUs (S1 File Table A) covers the range reported for MUs in rat medial gastrocnemius muscle with respect to all basic parameters [22].

The derived approximation Eqs (1–4) are based on 30 decomposed tetanic curves (Figs 3, 4 and 5 for dependencies). The most critical appears to be the relationship shown in Fig 4. The possible errors come from two sources: (1) the amplitudes of the single twitch and that of the first decomposed contraction are different for a MU depending on fatigue, potentiation, previous stimulations, etc. [23–25]; (2) the maximal tetanic force, especially for FF type MUs, may decrease due to fatigue and the estimation of this maximal force may also evoke an error. This explains why other angles—*α*2^*(j)*^—can significantly improve the prediction, and this was the reason for proposing the new, improved, power model by Eq (9).

Another reason for the impreciseness of the full prediction, using the calculated angle *α*1^*(j)*^ (Table 1), is that the predicted values of *F*^*pr*^_*tetmin*_^*(j)*^*(i)* are slightly different from the actual force levels at which the successive contractions start. Each of them starts with a delay of *T*_*lead*_^*(j)*^*(i)* (Fig 6). This lead time is not known in advance. It can be considered for the further improvement of the algorithm taking its value for the first contraction, i.e. *T*_*lead*_^*(j*)^(1), which can be added to *t*_*IPI(n)*_ in Eq (8).

The input data used for approximations were the results of decompositions of tetanic contractions recorded for a group of three types of MUs, for different stimulation patterns, with different mean frequencies, but at similar fusion indices and similar relative force levels. An open question remains: whether the approach would be suitable for force curves with considerably different fusion indices (for example, for nearly fused tetanic curves) or for stimulation with equal IPIs. As discussed above, the derived equations might be not applicable for MUs of other rat muscles or for muscles in other species. Nevertheless, one may expect that the equations for force modeling in other muscles would be based on a similar main idea when matched to specific databases for these muscles. Irrespective of the above restrictions, the proposed new approach for predicting the successive twitch-like responses fusing into tetanic contraction has a general character, due to the fact that the same algorithm can be used for all MU types within a muscle. It is also important that, with a progress in motor units’ research, the described model could be further suitably expanded by adding new modules related to the non-linearity in the summation of forces of many MUs, to potentiation observed in fast MUs during their long-lasting activity and to fatigue in fast MUs. At present, there is lack of sufficient data concerning these phenomena.

## Supporting Information

S1 File The six parameters of all motor units used in this study allow to graphically present each individual (or first decomposed) twitch using the analytical function given in Raikova et al. (2008). They constitute the database for the prediction algorithm presented in the paper (S1 File Table A). S1 File Table B presents examples of the used stimulation patterns, i.e. the IPIs between the subsequent 41 pulses. S1 File Table A. The basic parameters used when the approximation was built and the reconstruction was made (for explanation, see Fig 2). Consecutive rows present data for the 10 slow MUs, the 10 FR MUs and the 10 FF MUs used as an input database. The last three rows show the data for the three additional MUs of three types (S, FR and FF), which were used for verification of the approach. The 10 MUs of each type are listed in order of increasing forces of the first twitch, *F*_*max*_^*(j)*^*(1)*. S1 File Table B. Interpulse intervals for stimulation patterns used for experimental tetani shown in Fig 7 and Fig 8 IPI are given in milliseconds.
(DOC)
