# Supplementary material for: A General Mathematical Algorithm for Predicting the Course of Unfused Tetanic Contractions of Motor Units in Rat Muscle
Source: PLoS One. 2016 Sep 13;11(9):e0162385. doi: 10.1371/journal.pone.0162385 (PMC5021327; doi:10.1371/journal.pone.0162385)
Supplement: S1 File — They constitute the database for the prediction algorithm presented in the paper (S1 File Table A). S1 File Table B presents examples of the used stimulation patterns, i.e. the IPIs between the subsequent 41 pulses. S1 File Table A. The basic parameters used when the approximation was built and the reconstruction was made (for explanation, see Fig 2). Consecutive rows present data for the 10 slow MUs, the 10 FR MUs and the 10 FF MUs used as an input database. The last three rows show the data for the three additional MUs of three types (S, FR and FF), which were used for verification of the approach. The 10 MUs of each type are listed in order of increasing forces of the first twitch, Fmax(j)(1). S1 File Table B. Interpulse intervals for stimulation patterns used for experimental tetani shown in Fig 7 and Fig 8 IPI are given in milliseconds. (DOC) [file pone.0162385.s001.doc]

Supporting information

**S1 Table A.** The basic parameters used when the approximation was built and the reconstruction was made (for explanation, see Fig 2). Consecutive rows present data for the 10 slow MUs, the 10 FR MUs and the 10 FF MUs used as an input database. The last three rows show the data for the three additional MUs of three types (S, FR and FF), which were used for verification of the approach. The 10 MUs of each type are listed in order of increasing forces of the first twitch, *F_max_^(j)^(1)*.

| ***j*** | ***MU type*** | ***T_lead_^(j)^(1)*** | ***T_hc_^(j)^(1)*** | ***T_c_^(j)^(1)*** | ***T_hr_^(j)^(1)*** | ***T_tw_^(j)^(1)*** | ***F_max_^(j)^(1)*** | ***F_mftf_^(j)^*** |
| --- | --- | --- | --- | --- | --- | --- | --- | --- |
| 1 | S1 | 3.0 | 9.5 | 20.0 | 45.0 | 100 | 3.0 | 44.6 |
| 2 | S2 | 2.8 | 9.2 | 27.0 | 58.0 | 160 | 3.3 | 32.0 |
| 3 | S3 | 4.3 | 11.5 | 28.0 | 62.0 | 140 | 3.4 | 45.0 |
| 4 | S4 | 2.7 | 9.3 | 27.0 | 63.0 | 160 | 3.4 | 32.0 |
| 5 | S5 | 4.5 | 8.0 | 24.0 | 54.0 | 120 | 5.1 | 63.8 |
| 6 | S6 | 4.0 | 9.0 | 22.0 | 54.0 | 110 | 5.3 | 75.0 |
| 7 | S7 | 4.6 | 9.4 | 33.0 | 78.0 | 160 | 6.5 | 55.6 |
| 8 | S8 | 4.5 | 8.7 | 24.0 | 61.0 | 150 | 6.5 | 61.2 |
| 9 | S9 | 4.5 | 8.5 | 24.0 | 55.0 | 120 | 6.5 | 69.4 |
| 10 | S10 | 4.5 | 10.4 | 36.0 | 82.0 | 170 | 7.8 | 67.1 |
| 11 | FR1 | 3.4 | 5.6 | 18.5 | 43.0 | 160 | 21.0 | 79.8 |
| 12 | FR2 | 2.7 | 4.5 | 14.0 | 26.9 | 80 | 23.0 | 154.8 |
| 13 | FR3 | 2.6 | 5.2 | 13.5 | 28.4 | 160 | 23.7 | 115.0 |
| 14 | FR4 | 3.2 | 5.0 | 14.3 | 33.0 | 160 | 26.6 | 104.0 |
| 15 | FR5 | 2.9 | 5.9 | 16.7 | 36.5 | 80 | 38.9 | 201.3 |
| 16 | FR6 | 4.3 | 5.1 | 14.0 | 29.5 | 67 | 56.0 | 140.0 |
| 17 | FR7 | 4.3 | 4.6 | 13.5 | 27.5 | 65 | 70.5 | 179.0 |
| 18 | FR8 | 2.9 | 5.3 | 14.7 | 30.6 | 80 | 78.0 | 275.3 |
| 19 | FR9 | 2.9 | 5.1 | 13.8 | 29.0 | 80 | 126.7 | 408.0 |
| 20 | FR10 | 3.1 | 4.6 | 13.0 | 29.5 | 65 | 128.5 | 318.0 |
| 21 | FF1 | 3.1 | 4.8 | 13.4 | 25.4 | 60 | 22.7 | 85.8 |
| 22 | FF2 | 3.3 | 5.3 | 14.5 | 29.6 | 150 | 60.5 | 210.0 |
| 23 | FF3 | 3.0 | 5.6 | 15.0 | 31.0 | 160 | 64.5 | 210.0 |
| 24 | FF4 | 3.0 | 7.0 | 18.5 | 38.0 | 80 | 84.3 | 324.0 |
| 25 | FF5 | 3.4 | 5.2 | 14.0 | 29.0 | 78 | 91.3 | 185.0 |
| 26 | FF6 | 2.8 | 5.3 | 13.7 | 29.2 | 70 | 97.6 | 158.7 |
| 27 | FF7 | 4.0 | 5.1 | 13.8 | 26.5 | 49 | 129.5 | 275.0 |
| 28 | FF8 | 4.5 | 6.4 | 23.5 | 57.5 | 140 | 151.0 | 307.0 |
| 29 | FF9 | 4.3 | 4.9 | 13.0 | 31.0 | 69 | 175.0 | 369.0 |
| 30 | FF10 | 3.7 | 5.6 | 17.1 | 42.0 | 80 | 253.5 | 546.0 |
| 31 | S11 | 3.0 | 7.0 | 24.0 | 50.0 | 135 | 5.5 | 26.9 |
| 32 | FR11 | 2.6 | 5.2 | 13.5 | 28.4 | 160 | 23.7 | 115.0 |
| 33 | FF11 | 4.0 | 5.0 | 13.9 | 36.0 | 84 | 194.5 | 410.0 |

**S1 Table B.** Interpulse intervals for stimulation patterns used for experimental tetani shown in Fig 7 and Fig 8 IPI are given in milliseconds

**No IPI1 IPI2 IPI3 IPI4 IPI5 IPI6**

1 0 0 0 0 0 0

2 88 37 22 42 14 29

3 49 38 27 67 16 36

4 47 40 15 67 28 22

5 86 36 17 64 19 36

6 88 25 36 50 25 22

7 85 24 35 71 27 36

8 58 22 37 33 24 19

9 38 30 37 40 25 30

10 54 16 27 65 19 44

11 31 29 13 43 11 22

12 32 37 27 26 14 43

13 84 41 35 42 22 19

14 62 38 18 53 12 19

15 48 19 17 65 24 42

16 67 23 21 45 18 24

17 36 28 17 71 12 27

18 61 31 14 65 25 44

19 88 22 29 46 13 23

20 57 38 33 65 15 40

21 48 30 24 58 28 19

22 72 22 23 74 20 35

23 43 44 32 72 25 42

24 72 20 32 29 21 35

25 36 37 21 44 15 25

26 71 20 18 45 28 37

27 66 32 33 35 20 26

28 84 38 29 33 15 33

29 61 37 14 30 26 36

30 57 35 21 37 21 27

31 48 43 28 42 26 20

32 79 38 33 61 18 19

33 50 38 15 66 14 43

34 34 23 29 53 22 30

35 54 22 17 42 29 30

36 39 40 23 29 20 30

37 36 23 26 28 15 18

38 75 23 23 59 16 29

39 83 22 13 47 26 44

40 48 17 36 56 12 27

41 85 22 33 40 20 18
